# The relationship professional commitment and ethics with patient rights: a cross-sectional descriptive study

**DOI:** 10.1186/s12910-024-01084-2

**Published:** 2024-08-02

**Authors:** Sara Mohammadnejad, Afsaneh Raiesifar, Zoleikha Karamelahi, Razhan Chehreh

**Affiliations:** 1 Instructor of Geriatric Nursing, School of Midwifery and Nursing, Ilam University of Medical Sciences, Ilam, Iran; 2 Student Of Medical Ethics, School Of Medical, Shahid Beheshti University Of Medical Sciences, Tehran, Iran; 3 School of Midwifery and Nursing, Ilam University of Medical Sciences, Ilam, Iran; 4 Clinical Research Development Unit, 5 Azar Hospital, Golestan University of Medical Sciences, Gorgan, Iran; 5 School of Midwifery and Nursing, Ilam University of Medical Science, Ilam, Iran., Ilam, Iran

**Keywords:** Professional Commitment, Professional ethics, Patient right

## Abstract

**Background:**

Ethical behavior of health workers is an important part of health services. The aim of the present study was to determine the relationship between ethics and professional commitment and its relationship with the level of respect for patient rights in medical students.

**Material & methods:**

A cross-sectional descriptive study was conducted with the participation of nursing, midwifery and emergency medicine students of Ilam University of Medical Sciences. Sampling was done by stratified random method. The data was collected using Demographic, Professional Commitment, Professional ethics and a researcher made questioner on compliance with patient rights questionnaires.

**Results:**

300 students were participated. The results showed that the average score of professional ethics in middle school students is high (64.07 ± 8.01), the average score of professional commitment is also high (64.07 ± 8.01) and the score of respect for patient rights is also high (10.74). ± 83.46) was obtained. The professional ethics score it showed a positive and statistically significant relationship with the patient’s rights compliance score. only professional commitment is related to gender, but the average of all three variables in different age groups and the type of residence (dormitory, private home, etc.) have meaningful statistical difference.

**Conclusion:**

The findings of the study show that the level of ethics and professional commitment and respect for patient rights among nursing, midwifery and emergency medicine students was good. It is hoped that the results of this research will provide a basis for better planning for the development of knowledge and respect for patient rights among students.

**Supplementary Information:**

The online version contains supplementary material available at 10.1186/s12910-024-01084-2.

## Introduction

Ethical behavior of health workers is an important part of health services [1]. Healthcare personnel often face difficult ethical situations during their work, and these issues cover a wide range of clinical practice areas [2]. Providing ethical care is one of the important goals of various medical professions, and the performance based on ethics in the field of care improves the quality of health care [3]. Professionalism is also a basic concept in the fields of care, which is formed from the interaction of the person, the work environment and interpersonal communication. Professional commitment is either theoretically demonstrated or exemplified by faculty and others in clinical practice [4, 5].

Studies have shown that people working in health professions have a high professional commitment due to the opportunity given to them by this job to help others and provide valuable and meaningful service [6]. Professional commitment is formed gradually and there is a correlation between professional commitment in the college and the workplace. In other words, if the students of the Department of Medical Sciences have a higher professional commitment during their studies, they will have more commitment when entering the workforce [7]. Students of medical sciences are involved in professional ethics issues as an important part of health care workers. The role of ethics is essential in actions and behavior, in decisions and choices, in attitudes and communication. Professional ethics is a process of logical thinking to maintain and spread professional values in order to provide optimal conditions for the realization of the rights of beneficiaries with favorable social relations and is one of the basic pillars of professional education [8]. In a study, it has been stated that students recognize ethical issues in their profession and are sensitive to it [9]. Creating an embodied professional character in the field of professional ethics in medical sciences is not easy, because there is no single view of what medical professionalism entails [10]. Today, the need for professional graduates who are familiar with professional ethics and patient rights is more noticeable than ever, and nursing educators should increase the educational capabilities of students in order to increase the number of professional nurses [11].

The issue of professional ethics and patients’ rights has always been one of the concerns of healthcare professionals [12]. Respecting patients’ rights is the main priority of the country in the field of medical ethics. The important point is that patients’ rights are key pillars in determining clinical governance standards. One of the principles of professional ethics is to pay attention to the rights of patients and protect them. In fact, in addition to maintaining and promoting the health of patients, you should pay attention to their rights and make efforts to comply with them [13]. But the results of some studies have shown that in Iran, the awareness of the bill of rights of the patient and its observance by the medical staff is not in a good condition [14, 15]. Fazli et al. showed that the status of compliance with the charter of patient’s rights in educational-medical centers is favorable [16]. Another study showed that midwives’ knowledge of ethical laws and knowledge of patients’ rights are also effective in respecting the rights of pregnant women [17]. Patients’ rights are the expectations from healthcare institutions. In Richard’s study, it is stated that one of the challenges of educating students is whether they have a strong understanding of ethical issues. This understanding of ethical issues affects the relationship between the patient and the student [1, 8, 18]. Just as health care personnel are ethically committed to protecting the confidentiality of patient rights and respecting the principle of patient discretion as individuals involved in the patient treatment process, it is necessary for colleges to adopt guidelines regarding the levels of student-patient interaction. and to emphasize the education of patients’ rights in universities during their studies. Involvement of students in legal issues increases clinical skills, leadership skills and professional identity in them [19, 20]. Knowing the ethical code and the code of rights of patients by nursing and midwifery personnel will lead to better communication and support for patients and more patient satisfaction. On the other hand, this knowledge reduces medical costs and errors, and finally, professional independence and improving patient services. Also, considering the importance of respecting patient’s rights in students, identifying the influencing factors for them is particularly important. Therefore, the aim of the present study was to determine the relationship between ethics and professional commitment and its relationship with the level of respect for patient rights in medical students.

## Materials & methods

The current study is descriptive-cross-sectional. The statistical population of this research was nursing, midwifery and emergency medical students of Ilam University of Medical Sciences in the academic year of 2023. This research was conducted in two academic semesters in 2023. Three hundred nursing, midwifery and emergency medicine students of Ilam University of Medical Sciences participated in this study. Sampling was done by stratified random method in such a way that first each field of study was considered as a class and then samples were randomly selected from each class. According to the number of students studying in Ilam University of Medical Sciences and taking into account the possibility of dropping samples, the sample size was determined to be 300. The criteria for entering the study were: desire to participate in the study, studying at Ilam University of Medical Sciences, passing the professional ethics course. Also, unwillingness to cooperate and graduate students were the exit criteria. Three questionnaires were used to collect data.

### Demographic characteristics

Includes age, sex, year of study, field of study, grade point average, living with parents, place of residence and socio-economic level.

### Kleikman and Hining’s professional commitment questionnaire

This questionnaire consists of 15 items that are used to measure professional commitments. The scoring of the questionnaire is in the form of a 5-point Likert scale, for the options “completely disagree”, “somewhat disagree”, “neither agree nor disagree”, “somewhat agree” and “completely agree” with 1, 2, 3 ,4 and 5 points are considered. The minimum possible score will be 15 and the maximum will be 75. A score between 15 and 30 is low professional commitment, a score between 30 and 45 is medium professional commitment, and a score above 45 is high professional commitment. The validity and reliability of this questionnaire in Iran has been confirmed by Hosseini et al. (2015) with Cronbach’s alpha value of 0.90 [21].

### Kad Vazir’s professional ethics questionnaire

This questionnaire consists of 16 questions that measure professional ethics in 8 dimensions: responsibility (questions 1, 2), honesty (questions 3, 4), justice and fairness (questions 5, 6), loyalty (Questions 7, 8), superiority and competitiveness (Questions 9, 10), respect for others (Questions 11, 12), sympathy with others (Questions 13, 14) and respect for social values ​​and norms (Questions 15, 16). This questionnaire is in the form of a 5-point Likert scale, which is considered as 1, 2, 3, 4, 5 for the options very low, low, medium, high and very high. The scores obtained from the questions will be added together and considered as the total score of the exam. The higher the score, the higher the professional ethics and vice versa [22]. The validity and reliability of this questionnaire in Iran has been confirmed by Salimi and Khodaparast (2016) with Cronbach’s alpha value of 0.81 [23]. The data on compliance with patient rights was also collected by a researcher-made questionnaire based on the charter of patient rights, which included 19 questions. The order of scores 1, 2, 3, 4, 5 was considered. The sum of the points is 95 points, which according to the agreement, the level of compliance with the patient’s rights is defined as 95 to 70 good, 70 to 50 average, and below 50 poor. The present study was the result of a research project and obtained ethics approval from the code of ethics (IR.MEDILAM.REC.1402.096) from the ethics committee of Ilam University of Medical Sciences and obtaining the necessary permits, the objectives of the research were explained to the students and the confidentiality of the information was informed to them, and then informed consent was obtained from the students, then the number 300 questionnaires were completed by students electronically and the informed consent to participate in the study was obtained from the participants in the questionnaires. The questionnaire was entered into the PorsLine software, then its link was placed in the groups of students and completed by them. At the end, the data were analyzed using descriptive statistics, t-test, analysis of variance, Pearson correlation and regression.

## Results

43.3% of participating students were male. 46.3% of students were studying in nursing. The most frequent age group of students was 20–22 years old (Table 1).

The results showed that just the professional commitment average in male students was significantly higher than that of female students. The Kruskal-Wallis non-parametric test showed that the mean score of professional ethics, professional commitment, and respect for patient rights in nursing, midwifery and emergency medical students in different age groups does have a statistically significant difference. The average score of professional commitment in students who are independent from their families, professional ethics in students who have their own house with their family, and respect for patient’s rights in students who have a rented house with their family was significantly higher. The non-parametric U-Man-Whitney test also showed that the mean of professional ethics, professional commitment, and respect for patient rights in terms of living with parents in nursing, midwifery, and emergency medical students did not show a statistically significant difference. (Table 2)

The results showed that the average score of professional ethics in middle school students is high (64.07 ± 8.01), the average score of professional commitment is also high (64.07 ± 8.01) and the score of respect for patient rights is also high (10.74). ± 83.46) was obtained (Table 3).

Spearman’s correlation test was used to measure the linear relationship between professional ethics and professional commitment with respect for patient rights, and the results showed that there is no statistically significant relationship between the professional commitment score and the respect for patient rights in students, but the professional ethics score it showed a positive and statistically significant relationship with the patient’s rights compliance score. Also, there was a positive and statistically significant relationship between professional ethics and professional commitment (Table 4).

## Discussion

The results showed that the score of ethics and professional commitment was at a good level and the score of patient rights was also at a favorable level. Also, there was no statistically significant relationship between the professional commitment score and the patient’s rights compliance score in students, but the professional ethics score showed a positive and statistically significant relationship with the patient’s rights compliance score. Also, there was a positive and statistically significant relationship between professional ethics and professional commitment.

In line with the results of this study, in Razavi et al.‘s research (2023), the attitude score of dental students towards professional ethics was evaluated at a good level [24]. And the study of Jafari et al. (2019) showed the level of positive and favorable attitude of nursing and midwifery students towards professional ethics [25]. Shahbazzadeh et al.‘s study (2023) also showed the application of professional ethics at a desirable level among Ardabil nursing students [26]. Aliabadi et al.‘s study (2022) also showed a high level of knowledge and attitude towards professional ethics in nursing and medical students [21]. Opposite to these results, Mohammadi et al.‘s study (2016) evaluated nursing and midwifery students’ awareness of the principles of professional ethics at an average level and lower than that of operating room students [27]. Also, Dashti et al. (2017) also evaluated the students’ professional ethics score as average [28].

The findings of this study show that there is a significant relationship between professional ethics and respect for patients’ rights. In line with these results, the results of the study by Baqernia et al. (2022) comparing the level of awareness and compliance of nurses and midwives with patient rights and their professional ethics score showed that nurses are at an average level and midwives are at a high level. Also, there was a significant statistical relationship between the score of professional ethics and respect for patient rights between both groups [29]. Arab Ameri et al.‘s study (2022) in a review of professional values ​​among students showed that the most important component from the students’ point of view was keeping the patient’s secrets [30]. Mohammadi et al.‘s study (2015) also showed a significant and effective relationship between moral sensitivity and respect for patient’s rights among the studied nurses [31].

As the review of the literature showed, in various studies, the score of professional ethics among medical and paramedical students was medium and high. This result is influenced by the field of study, the sample size and the type of questionnaires. It is necessary to maintain the favorable current situation in medical and paramedical student by identifying and strengthening related factors. Also, the positive and significant relationship between professional ethics and patient rights was fully confirmed. Considering the confirmation of the relation between the two concepts of professional ethics and respect for patient’s rights, by strengthening each one, the other can be promoted. For example, holding training courses related to the knowledge of professional ethics can lead to an increase in students’ compliance with patients’ rights and vice versa.

In the present study, the professional commitment score was also evaluated at a favorable level, but no significant relationship was found between patient rights and professional commitment. In line with the results of the present study, Latifi et al. (2023) showed in their study that the average score of professional commitment in medical students is at an optimal level [32]. Zhao et al. (2022) also evaluated the professional commitment of nursing students before the internship period more than during the internship period and stated that in addition to demographic characteristics, the stress during the internship period can also lead to a decrease in professional commitment [33]. Contrary to the findings of the present study, it has been shown in a study that the professional commitment score of nursing students is at a low level and requires serious intervention [34]. Linan et al. (2021) also evaluated the level of professional commitment among Chinese nursing students as low [7]. The study of Rafiei et al. (2019) showed that the professional commitment of nurses was at a desirable level and the professional commitment of midwives was at a very good level, and there was a statistically significant relationship between professional commitment and awareness and compliance with the charter of patient rights among the studied nurses and midwives. These findings were not consistent with the results of the present study [35].

In general, the review of the literature in the field of professional commitment showed different results, in Iranian studies, the level of professional commitment of students was at a favorable level, but in studies of other countries, favorable conditions have not been reported. Part of which can be caused by the difference in the curriculum of the fields in different countries. It is very important to mention that in many countries, professional ethics is included as a subject in the educational curriculum and students are introduced to related concepts, but professional commitment is perhaps less considered in the educational course. Also in most study, professional commitment has been evaluated in graduates and employees of medical sciences, and students have been evaluated less. Therefore, in order to make a correct judgment about the current situation of students’ professional commitment, more studies with a larger sample size are necessary in this regard.

The present study showed a good situation regarding the observance of patient rights among students. In this regard, Khawajeh Ahmadi et al. (2015) evaluated the performance of nursing students in protecting the privacy of the patient’s rights at a favorable level [36]. In Mohammadi et al.‘s study (2015), the level of respect for patients’ rights in nurses was reported to be favorable, and the most respected dimensions were respect for the patient and respect for his privacy [37]. In the study of Kaya et al. (2016), the majority of nursing students evaluated human dignityد in the first place and justice in the second and third place, and the average score of nursing profession values was reported to be very high among them [38]. Rafiei et al.‘s study (2019) showed that the awareness and observance of the patient’s rights charter was average in nurses and high in the group of midwives [35].

Patient rights are one of the most sensitive and important areas in medical education, and there is always a fear that the patient’s rights will be violated during education of students at the bedside. However, based on the searches, studies on patient rights compliance have been done mostly in medical staff, and less attention has been paid to examining patient rights compliance in medical students, so more studies are needed to make a correct judgment about the level of attention and compliance of patients’ rights in students in different countries.

Regarding the relationship between demographic variables and the main variables of the present study, the findings showed that only professional commitment is related to gender, but the average of all three variables in different age groups and the type of residence (dormitory, private home, etc.) have meaningful statistical difference. In line with this study, Latifi et [32] also showed that there is a significant statistical relationship between age and academic semester with professional commitment, but there is no significant statistical relationship between other variables, including gender. Hoseinialiabadi study also showed a significant and positive relationship between students’ age and their knowledge and attitude regarding professional ethics, although other demographic variables did not have a statistically significant relationship with students’ professional ethics, which was different from the present study [21]. In Jafari et al.‘s study, there was no statistically significant relationship between midwifery and nursing students’ attitudes towards professional ethics and age, gender, field of study, and marital statuss [25]. The study of Shahbazzadeh in Ardabil showed that there is a statistically significant relationship only between the variables of interest in the field of nursing and the department type of work with the professional ethics [26]. In Mohammadi et al.‘s study, there was a statistically significant relationship between moral sensitivity and nurses’ attitude towards respecting patient’s rights and the age and number of years of service of nurses, which was different from the present study [37].

In general, studies have shown different results in terms of the relationship between variables of commitment and professional ethics and respect for patient rights with demographic variables, which can be caused by the diversity of participants, the study method and the tools used; Therefore, the need for more research in this area is felt.

## Conclusion

In general, the findings of the study show that the level of ethics and professional commitment and respect for patient rights among nursing, midwifery and emergency medicine students was good, and there was a significant relationship between professional ethics and respect for patient rights and professional commitment. Respecting the patient’s rights is one of the most important pillars of providing care. Considering the confirmation of the relation between the two concepts of professional ethics and respect for patient’s rights, by strengthening each one, the other can be promoted. For example, holding training courses related to the knowledge of professional ethics can lead to an increase in students’ compliance with patients’ rights and vice versa. Due to the low score of professional commitment in many studies it is also necessary to explain the concept of professional commitment to students more during the education course and to familiarize students with its dimensions and importance. Also, considering the importance of respecting patient’s rights in clinical education and the limitations of the studies conducted in this field, it is necessary to conduct more studies regarding the status of respecting patient’s rights and related factors in students of various fields of medical sciences.

Sampling from one center was one of the limitations of the present study, which was partially solved by random sampling.

**Table 1:** Demographic characteristics of nursing, midwifery and emergency medicine students

variable	Variable level	Frequency	Percent
sex	Male	130	43.3
	Female	170	56.7
Field of Study	Midwifery	96	32
	Nursing	139	46.3
	Medical Emergency	65	21.7
Education Year	First	123	41
	Second	38	7/12
	Third	68	7/22
	Fourth	71	7/23
Age	20 − 18	67	3/22
	22 − 20	111	37
	24 − 22	86	7/28
	24=<	36	12
Residency	Dormitory	173	7/57
	Private House Family	122	7/40
	Rented House Family	2	7/0
	Independent Form The Family	3	1
Academic average	14>	24	8
	99/15 − 14	118	3/39
	99/17 − 16	148	3/49
	20 − 18	10	3/3
Living with parents	Yes	271	3/90
	No	29	7/9
Socio-economic level	Low	36	12
	Medium	183	61
	Good	68	7/22
	Excellent	13	3/4

**Table 2:** Comparing the average response variables according to demographic characteristics

	Professional commitment	Professional ethics	Respecting the patient’s rights
Gender			
**Male**	89/7 ± 35/51	99/8 ± 18/64	66/11 ± 18/82
**Female**	67/4 ± 54/50	20/7 ± 98/63	90/9 ± 43/84
**P. value**	008/0	43/0	15/0
Age group			
**20 − 18**	11/5 ± 55/51	18/8 ± 28/66	62/9 ± 82
**22 − 20**	02/6 ± 83/64	50/6 ± 83/64	02/10 ± 81/84
**24 − 22**	89/6 ± 48/48	29/8 ± 80/61	78/12 ± 53/83
**24=<**	49/5 ± 31/54	87/9 ± 63	23/9 ± 81/81
**P. value**	001/0>	002/0	001/0>
Residence			
**Dormitory**	04/6 ± 328/51	63/7 ± 20/64	58/10 ± 42/81
**Private House Family**	39/6 ± 12/50	14/8 ± 47/64	79/10 ± 20/85
**Rented House Family**	44	55	87
**Independent Form The Family**	62	46	70
**P. value**	002/0	007/0	005/0
Living with parents			
**Yes**	35/6 ± 87/50	98/7 ± 18/64	01/11 ± 19/83
**No**	56/5 ± 10/51	32/8 ± 63	45/7 ± 93/85
**P. value**	36/0	48/0	40/0

**Table 3:** Mean and standard deviation of the studied variables

Variable	Maximum	Minimum	Mean ± Standard deviation
Professional commitment	64	16	27.6 ± 89.50
Professional Ethics	77	45	01.8 ± 0.064
Respecting the patient’s rights	95	50	10.74 ± 46.83

**Table 4:** The relationship between ethics and professional commitment with the level of patient rights compliance in students

Variable	Professional Ethics		Respecting the patient’s rights	
	Correlation value (*r*)	Meaningful (*P*)	Correlation value (*r*)	Meaningful (*P*)
**Professional commitment**	42.0	< 0.001	11.0	0.06
**Professional Ethics**	-	-	35.0	001.0>

### Electronic supplementary material

Below is the link to the electronic supplementary material.

Supplementary Material 1

## Data Availability

The data that support the findings of this study are available by request to correspond author.
