# Supplementary material for: The relationship professional commitment and ethics with patient rights: a cross-sectional descriptive study
Source: BMC Med Ethics. 2024 Aug 2;25:85. doi: 10.1186/s12910-024-01084-2 (PMC11295435; doi:10.1186/s12910-024-01084-2)
Supplement: Supplementary file 1 — Supplementary Material 1 [file 12910_2024_1084_MOESM1_ESM.pdf]

Patient rights compliance questionnaire

**Area 1 (optimal receipt of health services)**	**I completely disagree**	**I disagree**	**I have no opinion**	**I agree**	**I completely agree**
1-The patient has the right to receive a service that is worthy of human dignity and with respect for his values, cultural and religious beliefs.					
2-The patient has the right to receive a service that is free from any discrimination, including ethnic, cultural, religious, type of disease and gender.					
3-The patient has the right to receive a service that is based on the coordination of the elements of care, including prevention, diagnosis, treatment and rehabilitation.					
4-The patient has the right to receive a service that provides all basic and necessary amenities and is far from imposing unnecessary pain and suffering.					
**Area 2 (awareness of information in a favorable way and to a sufficient extent)**					
1-The information received by the patient should include the predictable criteria and costs of the hospital, including medical and non-medical services, insurance criteria and the introduction of support systems at the time of admission.					
2-The information received by the patient should include the name, responsibility and professional rank of the members of the medical group responsible for providing care, including doctors, nurses and students, and their professional relationship with each other.					
3-The information received by the patient should include all measures that are research in nature.					
4-The information received by the patient should include all the information recorded in his clinical file, access to the image of the file and request to correct the errors contained in					

Patient rights compliance questionnaire

it.					
**Area 3 (respecting the patient's right to make decisions and choose freely in receiving health services):**					
1-The patient can choose a doctor and a health service provider within the framework of the criteria.					
2-The patient has the right to participate or not to participate in any research.					
3-The patient has the right to accept or reject the proposed treatments after being aware of the possible side effects of accepting or rejecting them, except in cases of suicide or cases where refusing treatment puts another person at serious risk.					
4-The patient has the right to choose and ask the opinion of the second doctor as a consultant.					
**Area 4 (Providing health services to the patient based on respect for the patient's privacy)**					
1-Compliance with the principle of confidentiality regarding all information related to the patient is mandatory, except in cases where the law makes an exception.					
2-The patient has the right to have a trusted person with him during the diagnostic process, including examinations.					
3-The patient's privacy must be respected in all stages of care, both diagnostic and therapeutic.					
4-Only the patient and the treatment group and the authorized persons on behalf of the patient and the authorized persons according to the law can have access to the information.					
**Area 5 (the patient's right to access an efficient complaint handling system)**					
1-Patients have the right to be informed of the procedure and results of their complaints.					

Patient rights compliance questionnaire

2-The damage caused by the error of the health service providers must be compensated in the shortest possible time after investigation and proof according to the regulations.					
3-Every patient has the right to complain to the relevant authorities in case of violation of their rights based on the charter of patient's rights, without disturbing the quality of receiving health services.					
